# Lower Cambrian polychaete from China sheds light on early annelid evolution

**DOI:** 10.1007/s00114-015-1285-4

**Published:** 2015-05-28

**Authors:** Jianni Liu, Qiang Ou, Jian Han, Jinshu Li, Yichen Wu, Guoxiang Jiao, Tongjiang He

**Affiliations:** Early Life Institute, State Key Laboratory of Continental Dynamics, Department of Geology, Northwest University, Xi’an, 710069 China; Early Life Evolution Laboratory, State Key Laboratory of Biogeology and Environmental Geology, China University of Geosciences, Beijing, 100083 China

**Keywords:** Polychaete, Annelida, Guanshan Biota, Lower Cambrian

## Abstract

We herein report a fossilized polychaete annelid, *Guanshanchaeta felicia* gen. et sp. nov., from the Lower Cambrian Guanshan Biota (Cambrian Series 2, stage 4). The new taxon has a generalized polychaete morphology, with biramous parapodia (most of which preserve the evidence of chaetae), an inferred prostomium bearing a pair of appendages, and a bifid pygidium. *G. felicia* is the first unequivocal annelid reported from the Lower Cambrian of China. It represents one of the oldest annelids among those from other early Paleozoic Lagerstätten including Sirius Passet from Greenland (Vinther et al., Nature 451: 185–188, 2008) and Emu Bay from Kangaroo island (Parry et al., Palaeontology 57: 1091–1103, 2014), and adds to our increasing roll of present-day animal phyla recognized in the early Cambrian Guanshan Biota. This finding expands the panorama of the Cambrian ‘explosion’ exemplified by the Guanshan Biota, suggesting the presence of many more fossil annelids in the Chengjiang Lagerstätte and the Kaili Biota. In addition, this new taxon increases our knowledge of early polychaete morphology, which suggests that polychaete annelids considerably diversified in the Cambrian.

**Electronic supplementary material:**

The online version of this article (doi:10.1007/s00114-015-1285-4) contains supplementary material, which is available to authorized users.

## Introduction

Polychaete annelids are predominantly marine invertebrates, including over 80 recent families. They adopt a variety of lifestyles from swimming near the surface of the water column to burrowing in sediment (Fauchald and Rouse 1997; Rouse and Pleijel 2001). However, fossils of polychaete annelids, particularly body fossils, are relatively rare because polychaetes consist mostly of soft-bodied tissue, which easily decays (Briggs and Kear 1993). The earliest known annelids date from the Early Cambrian Sirius Passet fauna (Conway Morris and Peel 2008; Vinther et al. 2011). Whole-body fossils of annelids have been reported from a number of Palaeozoic Lagerstätten, such as the Middle Cambrian Burgess Shale (Conway Morris 1979), the Lower Ordovician deposits of Morocco (Vinther et al. 2008), the Silurian Herefordshire Biota of England (Briggs et al. 1996; Sutton et al. 2001), the Lower Devonian Hünsruck Slate (Högström et al. 2009; Briggs and Bartels 2010), the Middle Devonian Arkona Shale of Ontario (Farrell and Briggs 2007), the Carboniferous Bear Gulch Biota of Montana (Schram 1979) and the Mazon Creek Biota of Illinois (Thompson 1979). A detailed review of whole-body polychaete annelid fossil record has been provided (e.g., Bracchi and Alessandrello 2005; Parry et al. 2014). Although whole-body fossil annelids have been reported from most Paleozoic Lagerstätten (Briggs and Kear 1993), reliable records of annelids remain unknown from the famous Chengjiang Lagerstätte as well as the Kaili Biota. Here, we report a new polychaete annelid from the Guanshan Biota (Luo et al. 2008), which we name *Guanshanchaeta felicia* gen. et sp. nov. The Guanshan Biota are slightly younger than the Chengjiang Lagerstätte and older than the Kali Biota. As a diverse and informative Burgess Shale-type fossil Lagerstätte, the Guanshan Biota has yielded more than ten fossil groups: arthropods, brachiopods, sponges, eocrinoid echinoderms, cnidarians, hyolithids, vetulicolians, paleoscolecids, chancelloriids, anomalocaridids, lobopods, eldonoids and green algae (Hu et al. 2007; Liu et al. 2012; Steiner et al. 2012).

## Material and methods

A single specimen, ELI-GW-A001, was recovered from the Lower Cambrian (Cambrian Series 2, stage 4) Wulongqing Formation of the Gaoloufang section at Guangwei Village, Kunming, Yunnan Province. Details of the locality and stratigraphy of this formation are provided in Steiner et al. (2012). The specimen is deposited in the Early Life Institute of Northwest University, Xi’an, China. It was examined under a Leica Micro Kern Microscope and photographed using a dhs Microcam 3.3 camera through the ocular system of a Leica stereo microscope M125 (some photos were taken under 100 % ethanol). Drawings were made with a camera lucida on a Leica M125 Stereomicroscope. Measurements were directly made with a millimetre ruler. The photographs were first processed using PhotoShop 7.0, edited and collated in CorelDraw X4 and finally converted to TIFF format.

## Preservation

**Fig. 1:**
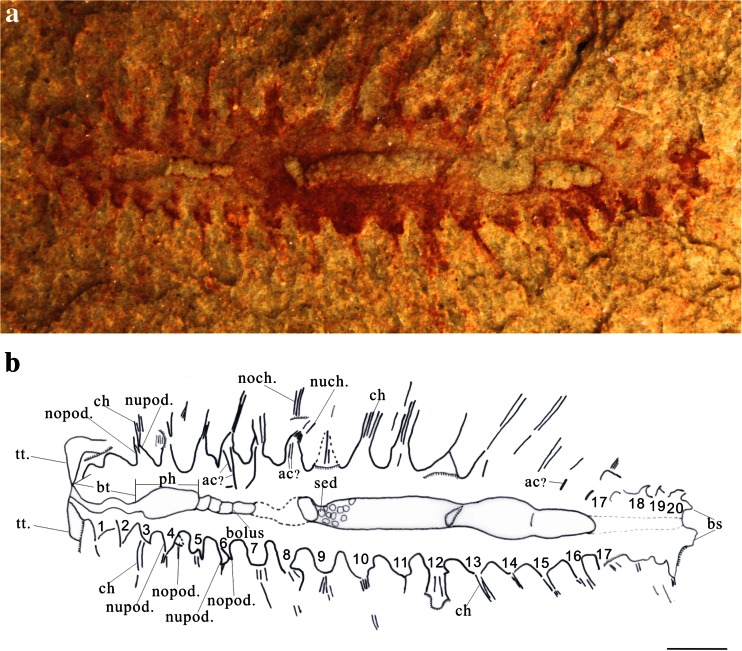
Holotype of *Guanshanchaeta felicia* gen. et sp. nov. ELI-GW-A001. **a** Complete specimen of ELI-GW-A001 photographed under ethanol. **b** Camera lucida of **a**, showing all characteristics of the specimen. Segments are numbered 1, 2, 3. *ac* acicula, *bs* bifid struture, *bt* buccal tube, *ch* chaetae, *nopod*. notopodium, *nupod*. neuropodium, *noch*. notochaetae, *nuch*. neurochaetae, *ph* pharynx, *sed*. sediment, *tt*. tentacle. Scale bar is 1 mm

**Fig. 2:**
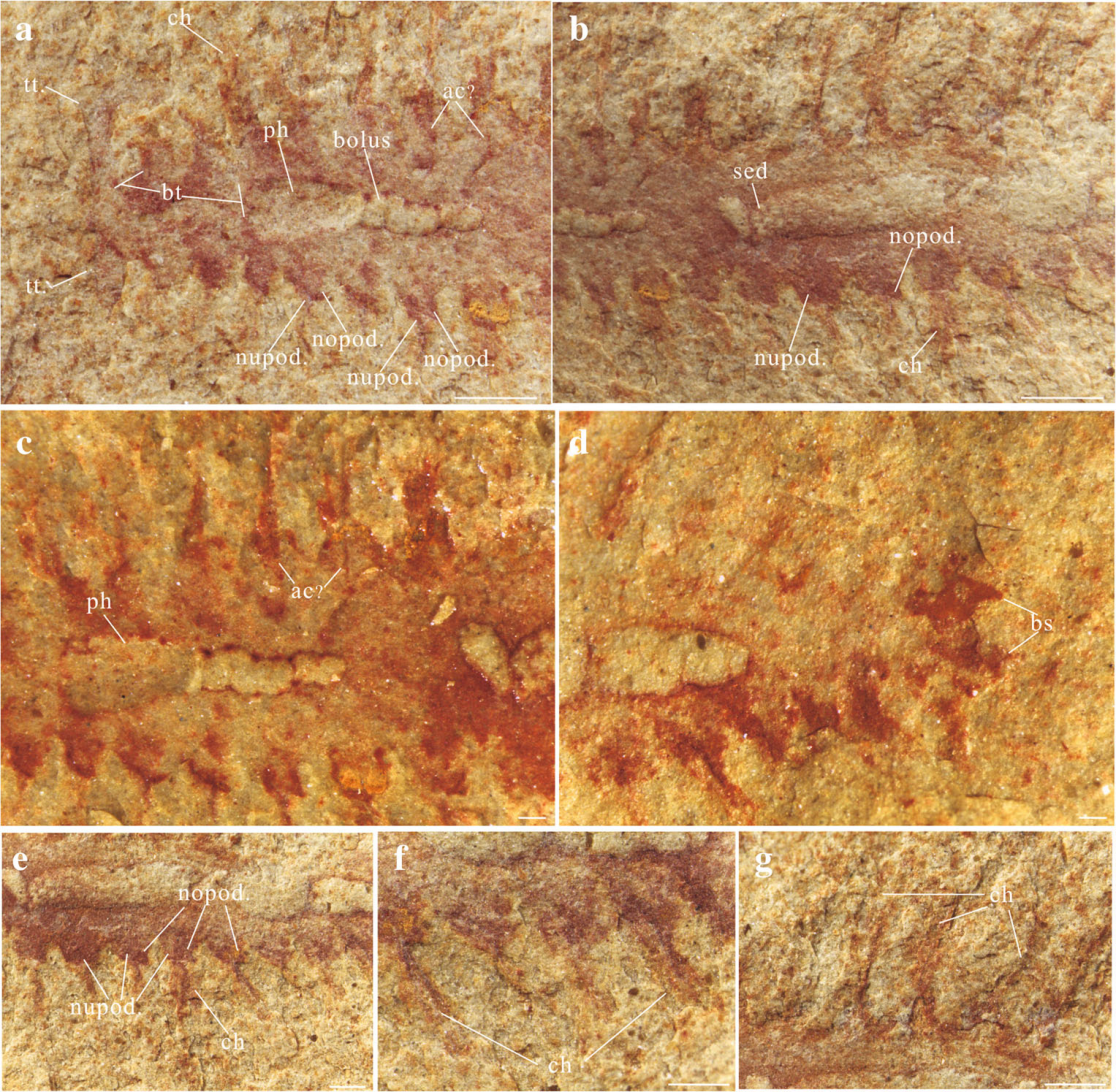
Holotype of *Guanshanchaeta felicia* gen. et sp. nov., ELI-GW-A001. **a** Enlargment of the anterior part of the holotype, showing the tentacles, parapodium and gut. **b** Enlargment of the middle part of the holotype; note the gut with sediment infill. **c** Enlargment of the anterior-middle part of the holotype photographed under ethanol, showing the aciculae. **d** Posterior part of the holotype photographed under ethanol, showing the bifid struture. **e** Enlargment of the holotype parapodia; note the notopodium and neuropodium. **f** Enlargment of chaetae on the left side of segments 12–15. **g** Enlargment of chaetae on the right side of segments 10–12. Abbrevations as in Fig. 1. Scale bar in **a**, **b** is 1 mm and in **c**–**g** is 0.2 mm

The specimen is preserved in pale-yellowish, thin-bedded silty mudstone, with two juveniles of the trilobite, *Palaeolenus douvillei* Mansuy, buried on the same lamella of the slab (supplementary Fig. 1a). The worm is flattened in parallel aspect, but the alimentary canal was filled with sediment and preserved with positive relief (Figs. 1 and 2a–c and supplementary Fig. 1), so it is likely that the cleavage plane is within the body, not at its dorsal or ventral surface. Fossilization of the animal, not least the cuticle and chaetae, is characterized by a thin film of diagenetic pyrite, which was subsequently weathered to iron dioxides, imparting the fossil a reddish appearance (Figs. 1 and 2 and supplementary Fig. 1). The parapodia are biramous, notopodia and neuropodia can be easily discerned because they are preserved on different lamellae (Figs. 1 and 2a, b, e and supplementary Fig. 1b). Given that the cleavage plane is within the body in the unique specimen, the dorsoventral orientation of the worm cannot be determined with confidence, but it is assumed to be at ventral view (and left and right are designated accordingly below).

### Systematic palaeontology

Phylum. Annelida Lamarck, 1809

Genus. *Guanshanchaeta* gen. nov.

Derivation of name. *Guanshan*—referring to the name of the Konservat-Lagerstätte, which yielded the fossil; chaeta—characteristic element of polychaete morphology.

Diagnosis. Slender, elongate body, about 1.3 cm long; head bears a pair of tentacles. Trunk of 20 setigerous segments, all with biramous parapodia. Each parapodia bearing a notopodium and a more distinct neuropodium. Chaetae long and simple, approximately five in a bundle. Cirri and branchiae are absent or not preserved. Straight gut apparently with prominent boluses (presumably gut contents).

Species. *Guanshanchaeta felicia* sp. nov.

Derivation of name. Latin *felicia*, good fortune, alluding to the rarity of the taxon.

Diagnosis. As for genus.

Description.

**Fig. 3:**
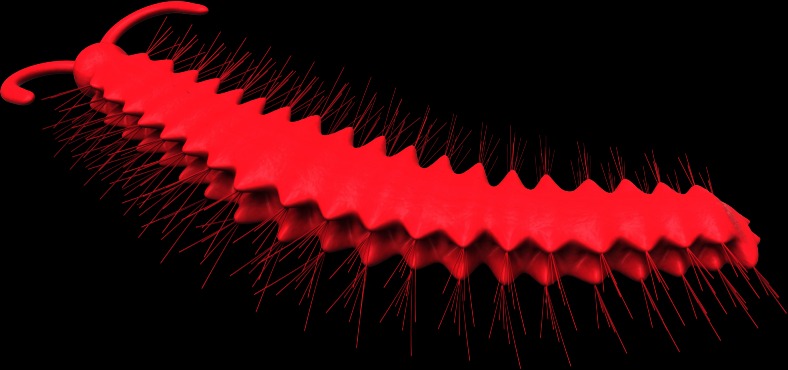
3D reconstruction of *Guanshanchaeta felicia* gen. et sp. nov.

The worm is bilaterally symmetrical and elongate, tapering both anteriorly and posteriorly (Figs. 1a and 3 and supplementary Figs. 1 and 2). It is approximately 1.3 cm long and about 0.5 cm wide at its widest point, including outstretched parapodia. The specimen is flattened in the parallel aspect. The body is divided into a head region with a pair of tentacles and a trunk, with laterally projecting parapodia. The boundary between the head and trunk is poorly defined. The head tapers anteriorly to a blunt termination, scarcely narrower than the rest of the body (approx. 1 mm in width, Figs. 1, 2a and 3 and supplementary Figs. 1b and 2), while its posterior margin lacks any clear boundary. From the anterior corners of the head, a pair of long, flat and smooth tentacles arise (Figs. 1, 2a and 3 and supplementary Figs. 1b and 2). The tentacles are recurved, and their distal parts are buried underneath the trunk (Figs. 1, 2a and 3 and supplementary Figs. 1b and 2). Thus, we cannot determine their length, but their exposed part is about 1 mm long and 0.3 mm wide. The anterior of the head is incompletely preserved; consequently, we could not observe the potential presegmental prosotmium and peristomium, cirri and mouth. However, from the anterior terminus, a buccal tube extends posteriorly into an expanded pharynx (Figs. 1 and 2a, c and supplementary Fig. 1b). The trunk consists of 20 homonomous segments. The trunk width increases posteriorly, reaching an acme at the ninth trunk segment, then tapering over the five posterior-most segments. Biramous parapodia arise along the lateral margins of the trunk (Figs. 1, 2a, b, e and 3 and supplementary Figs. 1 and 2). In accordance with the overall shape of the specimen, the size of the parapodia decreases both anteriorly and posteriorly. The neuropodium is comparatively large and sub-triangularly shaped and sometimes overlay the notopodium (Figs. 1 and 2a, b, e and supplementary Figs. 1b and 2). In segments 4, 5 and 6, only the right distal tips are present (Fig. 1). The neurochaetae are more distinct compared with the notochaetae. The neurochaetae are brush-like, arising from the tip of each neuropodium and diverging distally (Figs. 1 and 2e–g and supplementary Figs. 1b and 2). Each chaeta is long and robust. The number of chaetae per bundle is difficult to estimate precisely, but five chaetae are observed on the right bundle of segment 10 (Fig. 1). There are clear linear structures on some segments, particularly on the right side, which extend beyond the proximal part of the parapodia and into the body (Figs. 1 and 2a, c). We consider that these structures are either parapodial chaetae that have been shed and superimposed onto the body or remains of aciculae. The aciculae interpretation is consistent with that of the *Bundenbachochaeta* from Hunsrück (Briggs and Bartels 2010) in size and morphology. However, the presence of aciculae, a character of phylogenetic significance, has yet to be confirmed by additional specimens. The trunk terminates in a bifid structure (Figs. 1, 2d and 3 and supplementary Figs. 1 and 2), probably the postsegmental pygidium, which may have been adorned with a pair of pygidial cirri.

The most distinct internal feature of this specimen is a simple alimentary canal, which is well preserved and shows regional modifications (Figs. 1 and 2a, b and supplementary Fig. 1). Along the first three segments, there is a slim and thin tube, approximately 1.5 mm long and 0.2 mm wide (Figs. 1 and 2a and supplementary Fig. 1b), which is likely a buccal tube. The succeeding part (from the third to the fifth segments) is expanded and funnel-like and probably represents a pharynx (Figs. 1, and 2a and supplementary Fig. 1b). Whether this structure could evert into a proboscis, as observed in *Canadia spinosa* from the Burgess Shale (Conway Morris 1979), is difficult to discern. The pharynx is followed by a straight part with four internal boluses (from the fifth to the seventh segments, Figs. 1 and 2a and supplementary Fig. 1b), which are probably aggregations of undigested food particles. The next part (seventh to eigth segment) is smooth with no gut contents. Segments 9–17 consist of the most prominent part of the alimentary canal (Figs. 1 and 2b and supplementary Fig. 1b), where the gut is conspicuously filled with sediment (occupying about 50 % of the trunk width). The remainder of the alimentary canal is unclear; even the rim of the gut is obscure (Figs. 1 and 2d and supplementary Fig. 1b). The anus was presumably located at the end of the trunk as in other polychaetes.

## Discussion

### Affinities with extant polychaetes

Molecular evidence has demonstrated that clitellates are derived polychaetes and hence polychaetes are paraphyletic (Struck et al. 2007). Further phylogenomic analyses of various annelid taxa using amino acid positions suggests that this phylum can be largely divided into Errantia (Amphinomida, Phyllodocida, Orbiniidae, Eunicida, etc.) and Sedentaria (Clitellata, Canalipalpata, Echiura, Scolecida, etc.), with myzostomids and sipunculids positioned in the basal part of the tree (Struck et al. 2011). Primitive (stem group) annelids were probably errant, epibenthic polychaete having biramous parapodia with simple chaetae, and prostomial sensory appendages (Parry et al. 2014; Weigert et al. 2014). Crown-group annelids have been inferred to have evolved during Late Cambrian–Ordovician according to molecular and fossil evidence (Edgecombe et al. 2011; Erwin et al. 2011; Parry et al. 2014).

Therefore, taking into account the geochoronology of the Guanshan biota, *G. felicia* can be placed in the Polychaeta stem group of the Annelida based on its parapodia-like appendages with chaeta and the presence of head appendages. Nevertheless, its exact relationships within the polychaete annelids (and various fossils attributed to this group) are difficult to resolve.

### Affinities with fossil polychaetes

*G. felicia* is the first unequivocal record of an annelid polychaete from the Cambrian in China. Thus, a comparison between *G. felicia* and other Cambrian annelids is imperative. Based on this new finding, it would be helpful to construct a comprehensive cladogram of the Cambrian polychaetes. However, our cladistic experiments have failed to obtain a strict consensus tree (see Supplementary Table) for the following reasons: the morphology of the Cambrian polychaete taxa is remarkably diverse (Conway Morris 1979; Eibye-Jacobsen 2004), there are few convincing synapomorphies and very few apomorphies are unequivocally accepted by different authors who place their own interpretations on various characters (Conway Morris 1979; Eibye-Jacobsen 2004; Vinther et al. 2011). Thus, a robust polychaete phylogeny by cladistic analyses would not be feasible until more fossil specimens are available. Herein, we just provide comparisons of *Guanshanchaeta* with other Cambrian polychaetes on an individual basis.

Most of the Cambrian polychaetes have been discovered in the Canadian Burgess Shale fauna: *Burgessochaeta*, *Canadia*, *Insolicorypha*, *Peronochaeta* and *Stephenoscolex*. The overall appearance of *Guanshanchaeta* is dissimilar to any of these known polychaetes. However, the tentacles of *Guanshanchaeta* somewhat resemble those of *Canadia* and *Burgessochaeta* (Conway Morris 1979), although the latter are more evident and apparently more slender. Eibye–Jacobsen (Eibye-Jacobsen 2004) suggested two possibilities for the tentacles of Cambrian polychaetes: paired palps and lateral antennae. He concluded that the tentacles of *Canadia* and *Burgessochaeta* more likely represented palps than lateral antennae. Here, considering that the tentacles of *Guanshanchaeta* are similar in overall morphology, size and position to the Burgess Shale taxa (particularly *Burgessochaeta*), we also prefer to interpret the tentacles of *Guanshanchaeta* as paired palps.

The biramous parapodia of *Guanshanchaeta* markedly differ from the uniramous parapodia of *Peronochaeta*. Moreover, the parapodia of *Guanshanchaeta* are less distinctly shaped than those of *Insolicorypha*. The biramous parapodia of *Guanshanchaeta* are very simple, with almost no morphological differences between the notopodium and neuropodium. Their chaetal shape and structure is more akin to the equivalent structures of *Burgessochaeta* compared with the complex parapodia of *Canadia. Guanshanchaeta* shows almost no similarities with the disputed taxon *Wiwaxia* (Conway Morris 1979; Eibye-Jacobsen 2004).

Another famous Cambrian Lagerstätte that yields annelids is the Early Cambrian Sirius Passet fauna, from which *Phragmochaeta canicularis* (Conway Morris and Peel 2008) and *Pygocirrus butyricampum* (Vinther et al. 2011) have been reported. *Phragmochaeta* is incomplete (lacking a head), but its chaetae are distinct. The chaeta of *Guanshanchaeta* contains less information compared with those of *Phragmochaeta*, but the noto- and neurochaetae are similar in both taxa. In addition, the alimentary canal of both species contains prominent infillings. However, *Guanshanchaeta* lacks the musculature observed in *Phragmochaeta*.

*Pygocirrus* is also incomplete (lacking a head), but its pygidial cirri had not been reported in Cambrian annelids prior to Vinther et al. (2011). *Guanshanchaeta* terminates in a bifid structure, possibly representing the remnants of pygidial cirri or a bifid pygidium. Both *Guanshanchaeta* and *Pygocirrus* possess biramous parapodia, but the latter exhibits many more countable capillary chaetae.

In addition to the fossils from the Cambrian deposits, *Guanshanchaeta* is similar to *Bundenbachochaeta eschenbachensis* from the Lower Devonian Hunsrück Slate (Briggs and Bartels 2010). Both annelids are elongated and have biramous parapodia (with the neuropodia more distinct compared with the notopodia), a pair of appendages on the prostomium and possible aciculae. The trunks of both species terminate in a bifid structure.

### Evolutionary implications

The phylum Annelida is systematically and ecologically important. Annelid phylogeny currently focuses on the origins of segmentation, the coelom and β-chitinous chaetae. However, annelid evolution remains controversial. They were traditionally classified into two major groups—Polychaeta and Clitellata (largely Oligochaeta + Hirudinea). Based on anatomical features, Rouse and Fauchald (1997) further divided polychaetes into Scolecida and Palpata (Canalipalpata + Aciculata). However, this scheme has been rendered obsolete since most morphological and molecular analyses have demonstrated that Hirudinea is an ingroup of the Clitellata, which in turn is an ingroup of the Polychaeta (McHugh 1997; Nielsen 2012). Cladistic analyses based on morphological characters (Fauchald and Rouse 1997; Rouse and Fauchald 1997), molecular data (Rousset et al. 2007; Struck et al. 2007, 2008; Struck 2011) or a combination thereof (Zrzavý et al. 2009; Parry et al. 2014) all support the Clitellata group. However, more recent phylogenomic studies (Struck et al. 2011) support a traditional Errantia–Sedentaria grouping, which suggests the inclusion of Clitellata in the Sedentaria and, more intriguingly, proposes that the ancestral annelid probably possessed an errant polychaete body form, possessing biramous parapodia with simple chaetae, and prostomial sensory palps (cf. Parry et al. 2014; Weigert et al. 2014).

**Fig. 4:**
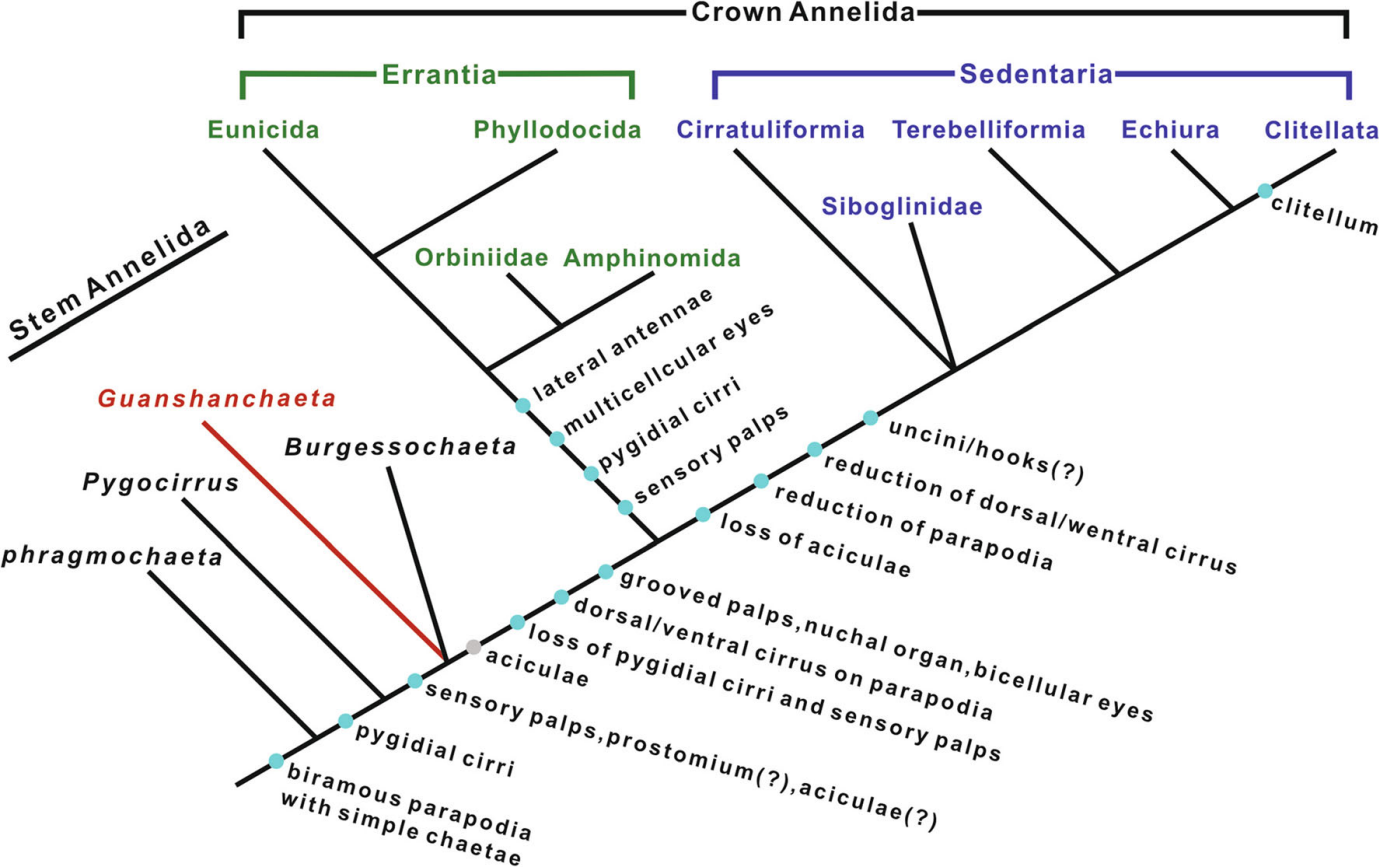
Hypothetical cladogram accounting for phylogenetic position of the Camrian *Guanshanchaeta* on the annelid stem lineage. Framework of annelid evolution is adapted from phylogenomic analyses by Struck et al. (2011) and review by Parry et al. (2014). *Cyan circles* denote acquisition or loss of synapomorphies; *grey circle* denotes acquisition of aciculae in case this character was not present in *Guanshanchaeta*. According to Struck et al. (2011), aciculae are a plesiomorphy of the clade grouping Errantia and Sedentaria rather than an apomorphy of Errantia (Vinther et al. 2011)

Polychaetes from the early Cambrian Sirius Passet fauna (*Phragmochaeta* and *Pygocirrus*) and the middle Cambrian Burgess Shale biota (*Burgessochaeta*, *Canadia*, etc.) have been assigned to the stem group (Conway Morris 1979; Conway Morris and Peel 2008; Vinther et al. 2011), with the early acquisition of key characters including sensory palps, parapodia, capillary chaetae and pygidial cirri. Our fossil evidence seems to support this supposition. We hypothesize that *Guanshanchaeta* resides in the annelid stem lineage and indicates the acquisition of some key morphological features such as sensory palps and perhaps prostomium and aciculae (Fig. 4). Accordingly to a robust annelid phylogeny constructed by molecular evidence (Struck et al. 2011), the last common ancestor of annelids possessed a pair of anterior grooved palps (functioned in both food gathering and sensory perception), bicellular eyes and nuchal organs as sensory organs, biramous parapodia with developed notopodium and neuropodium, and such chaetal types as aciculae (internalized supporting chaetae) and simple chaetae, but lacked other head or pygidial appendages (cf. Weigert et al. 2014). Among them, the biramous parapodia, simple chaetae and aciculae might have evolved in the Cambrian stem lineage (Fig. 4). However, pygidial cirri (see Fig. 2e and discussion in the text; Vinther et al. 2011) and solid sensory palps (see Fig. 2b and discussion; Eibye-Jacobsen 2004), which evolved in the stem group, might have been lost during the odyssey leading to crown annelids and secondarily acquired in the major Errantia branch, if the proposition based on amino acid sequence (Struck et al. 2011) is correct. Given the presence of aciculae in the new form, we are allowed to infer that a stem lineage represented by *Guanshanchaeta* innovated such internal supporting chaetae to increase their mobility. This feature may have been inherited by the Errantia (Fig. 4), but lost in Sedentaria (coupled with reduction of parapodia) by adaptation to a sedentary life mode (Struck et al. 2011; Struck 2011). In this scenario, aciculae evolved early in the history of this phylum, probably before the evolution of grooved palps for collecting food particles. In addition, we infer that the grooved feeding palps, dorsal/ventral cirrus on parapodia, nuchal organ and bicellular eyes are probably subsequent innovations, occurring during the evolutionary steps leading to the crown annelids.

## Conclusion

*G. felicia* is the first unequivocal annelid reported from the Lower Cambrian of China and one of the oldest annelids with head appendages. This taxon adds to the increasing roll of present-day animal phyla recognizable in the early Cambrian Guanshan Biota and helps clarify the sequence of some key apomorphies acquired during the early history of annelid oddyssey. Although phylogenetic conclusions based on fossil annelid sequences remain problematic, this finding indisputably expands the panorama of the Cambrian ‘explosion’. As reported by Beesley et al. (2000), the Cambrian was a period of considerable taxonomic diversification among the polychaetes.

## Electronic supplementary material

ESM 1 (DOC 4071 kb)

## References

[CR1] Beesley PL, Ross GJB, Glasby CJ (2000) Polychaetes and allies: the southern synthesis. Fauna of Australia. Vol. 4A Polychaeta, Myzostomida, Pogonophora, Echiura, Sipuncula. Melbourne CSIRO Publishing, Melbourne, Australia, pp 148–150

[CR2] Bracchi G, Alessandrello A (2005). Paleodiversity of the free-living polychaetes (Annelida, Polychaeta) and description of new taxa from the Upper Cretaceous Lagerstätten of Haqel, Hadjula and Al-Namoura (Lebanon). Mem Soc Ital Sci Nat Museo Civico Storia Naturale Milano.

[CR3] Briggs DEG, Bartels C (2010). Annelids from the Lower Devonian Hunsrűck Slate (Lower Emsian, Rhenish Massif, Germany). Palaeontology.

[CR4] Briggs DEG, Kear AJ (1993). Decay and preservation of polychaetes: taphonomic thresholds in soft-bodied organisms. Paleobiology.

[CR5] Briggs DEG, Siveter DJ, Siveter DJ (1996). Soft-bodied fossils from a Silurian volcaniclastic deposit. Nature.

[CR6] Conway Morris S (1979). Middle Cambrian polychaetes from the Burgess Shale of British Columbia. Phil Trans RSoc of Lond B.

[CR7] Conway Morris S, Peel JS (2008). The earliest annelids: Lower Cambrian polychaetes from the Sirius Passet lagerstätte. Peary land, north Greenland. Acta Pal Pol.

[CR8] Edgecombe GD, Giribet G, Dunn CW, Hejnol A, Kristensen RM, Neves RC, Rouse GW, Worsaae K, Sørensen MV (2011). Higher-level metazoan relationships: recent progress and remaining questions. Org Divers Evol.

[CR9] Eibye-Jacobsen D (2004). A reevaluation of *Wiwaxia* and the polychaetes of the Burgess Shale. Lethaia.

[CR10] Erwin DH, Laflamme M, Tweedt SM, Sperling EA, Pisani D, Peterson KJ (2011). The Cambrian conundrum: early divergence and later ecological success in the early history of animals. Science.

[CR11] Farrell Ú, Briggs DEG (2007). A pyritized polychaete from the Devonian of Ontario. Proc R Soc B.

[CR12] Fauchald K, Rouse G (1997). Polychaete systematics: past and present. Zool Scr.

[CR13] Högström AES, Briggs DEG, Bartels C (2009). A pyritized lepidocoleid machaeridian from the Lower Devonian Hunsrück Slate Germany. Proc R Soc B.

[CR14] Hu S-X, Luo H-L, Hou S-G, Erdtmann BD (2007). Eocrinoid echinoderms from the Lower Cambrian Guanshan Fauna in Wuding, Yunnan, China. Chin Sci Bull.

[CR15] Liu J-N, Ou Q, Han J, Zhang Z-F, He T-J, Yao X-Y, Fu D-J, Shu D-G (2012). New occurence of the Cambrian (stage 4, series 2) Guanshan Biota in Huize, Yunnan south China. Bull Geo.

[CR16] Luo H-L, Li Y, Hu S-X, Fu X-P, Hou S-G, Liu X-R, Chen L-Z, Li F-J, Pang J-Y, Liu Q (2008). Early Cambrian Malong fauna and Guanshan Fauna from eastern Yunnan China.

[CR17] McHugh D (1997). Molecular evidence that echiurans and pogonophorans are derived annelids. Proc Natl Acad Sci U S A.

[CR18] Nielsen C (2012). Animal evolution: interrelationships of the living phyla.

[CR19] Parry L, Tanner A, Vinther J (2014). The origin of annelids. Palaeontology.

[CR20] Rouse GW, Fauchald K (1997). Cladistics and polychaetes. Zool Scr.

[CR21] Rouse GW, Pleijel F (2001) Polychaetes. Oxford University Press, London.

[CR22] Rousset V, Pleijel F, Rouse GW, Erséus C, Siddall ME (2007). A molecular phylogeny of annelids. Cladistics.

[CR23] Schram FR (1979). Worms of the Mississippian Bear Gulch Limestone of central Montana, USA. Trans San Diego Soc Nat Hist.

[CR24] Steiner M, Hu S-X, Liu J-N, Keupp H (2012). A new species of *Hallucigenia* from the Cambrian stage 4 wulongqing formation of Yunnan (south China) and the structure of sclerites in lobopodians. Bull Geo.

[CR25] Struck TH (2011). Direction of evolution within Annelida and the definition of Pleistoannelida. J Zool Syst Evol Res.

[CR26] Struck TH, Schult N, Kusen T, Hickman E, Bleidorn C, McHugh D, Halanych KM (2007). Annelid phylogeny and the status of Sipuncula and Echiura. BMC Evol Biol.

[CR27] Struck TH, Nesnidal MP, Purschke G, Halanych KM (2008). Detecting possibly saturated positions in 18S and 28S sequences and their influence on phylogenetic reconstruction of annelida (lophotrochozoa). Mol Phylogenet Evol.

[CR28] Struck TH, Paul C, Hill N, Hartmann S, Hösel C, Kube M, Lieb B, Meyer A, Tiedemann R, Purschke G, Bleidorn C (2011). Phylogenomic analyses unravel annelid evolution. Nature.

[CR29] Sutton MD, Briggs DEG, Siveter DJ (2001). A three-dimensionally preserved fossil polychaete worm from the Silurian of Herefordshire England. Proc R Soc B.

[CR30] Thompson I (1979). Errant polychaetes (Annelida) from the Pennsylvanian Essex fauna of northern Illinois. Palaeontographica A.

[CR31] Vinther J, Roy PV, Briggs DEG (2008). Machaeridians are Palaeozoic armoured annelids. Nature.

[CR32] Vinther J, Eibye-Jacobsen D, Harper DAT (2011). An Early Cambrian stem polychaete with pygidial cirri. Bio Lett.

[CR33] Weigert A, Helm C, Meyer M, Nickel B, Arendt D, Hausdorf B, Santos SR, Halanych KM, Purschke G, Bleidorn C, Struck TH (2014). Illuminating the base of the annelid tree using transcriptomics. Mol Biol Evol.

[CR34] Zrzavý J, Říha P, Piálek L, Janouškovec J (2009). Phylogeny of Annelida (Lophotrochozoa): total-evidence analysis of morphology and six genes. Evol Bio.

