# Supplementary material for: Lower Cambrian polychaete from China sheds light on early annelid evolution
Source: Naturwissenschaften. 2015 May 28;102(5-6):34. doi: 10.1007/s00114-015-1285-4 (PMC4446521; doi:10.1007/s00114-015-1285-4)
Supplement: Supplementary file 1 — (DOC 4071 kb) [file 114_2015_1285_MOESM1_ESM.doc]

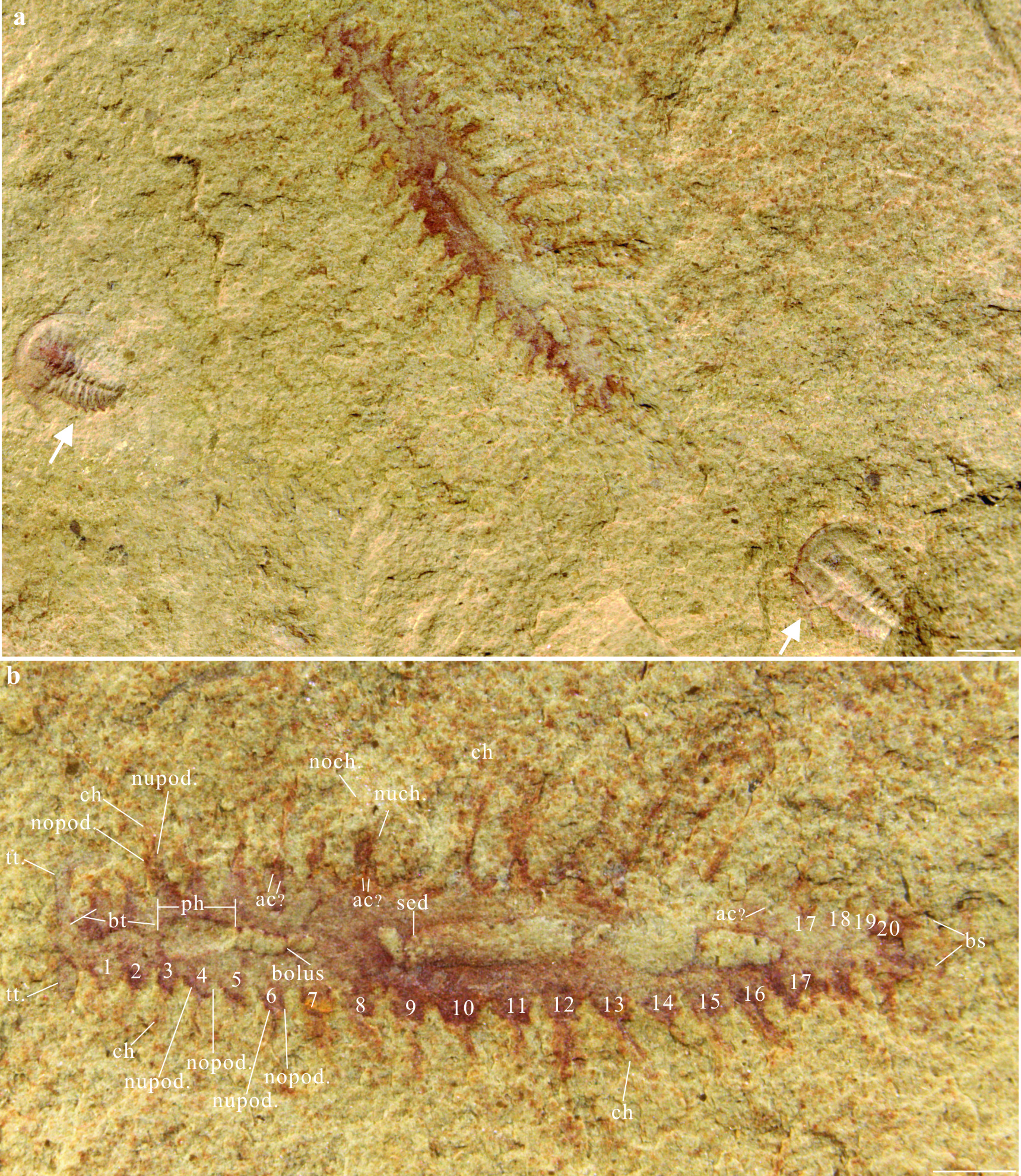
 **Supplementary Fig 1.** Holotype of *Guanshanchaeta* *felicia* gen. et sp. nov. ELI-GW-A001. **a** Complete specimen of ELI-GW-A001 with two trilobites, *Palaeonlenus douvillei* Mansuy (indicated by arrows). **b** Whole specimen of ELI-GW-A001. Segments are numbered 1, 2, 3. Abbrevations: ac, acicula; bs: bifid struture; bt: buccal tube; ch, chaetae; nopod., notopodium; nupod., neuropodium; noch. notochaetae; nuch. neurochaetae; ph, pharynx; sed. sediment; tt. tentacle. Scale bar is one millimetre.


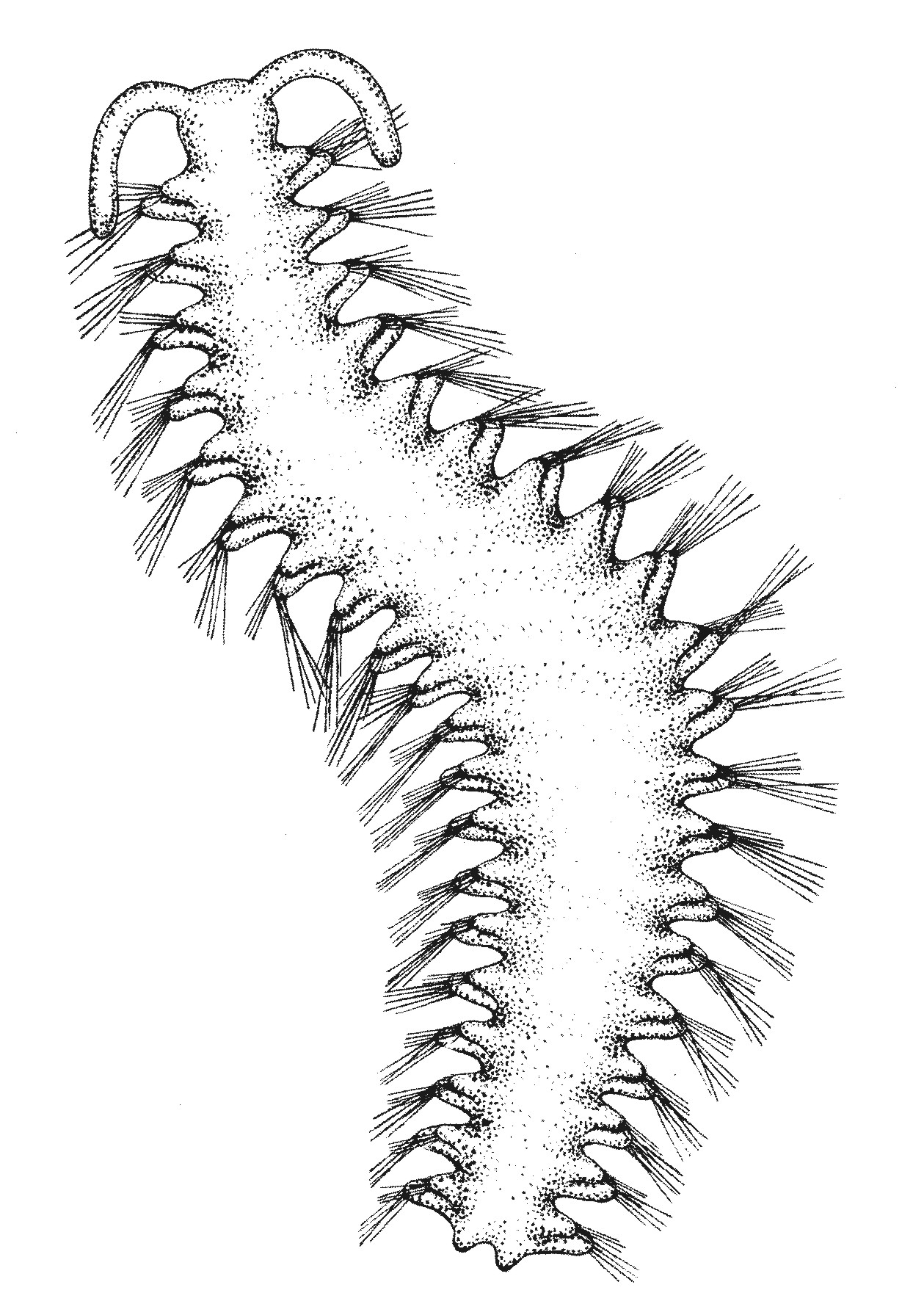


**Supplementary Fig 2.** Reconstruction of *Guanshanchaeta* *felicia* gen. et sp. nov.

**Supplementary Table**. Characters and character states used in cladistic analysis of Cambrian Polychaetes *Guanshanchaeta, Burgessochaeta, Canadia, Insolicorypha, Peronochaeta, Phragmochaeta, Pygocirrus,* Devonian polychaetae *Bundenbachochaeta* and Recent polychaetes. Characters are modified from Eibye-Jacobsen (2004).

1. Metamerism: 0, absent; 1, present

2. Palps: 0, absent; 1, present

3. Median antennae: 0, absent; 1, present

4. Lateral antennae: 0, absent; 1, present

5. Hypertrophied axial stomodaeum: 0, absent; 1, present

6 Proboscis: 0, absent; 1, present

7. Jaws: 0, absent; 1, present

8. Parapodia: 0, absent; 1, present

9. Compound chaetae: 0, absent; 1, present

10. Aciculae: 0, absent; 1, present

11. Dorsal cirri: 0, absent; 1, present

12. Ventral cirri: 0, absent; 1, present

13. Anterior dorsal and ventral cirri specialized as “tentacular” cirri: 0, absent; 1, present

14. Pygidial cirri: 0, absent; 1, present

| Character | 1 | 2 | 3 | 4 | 5 | 6 | 7 | 8 | 9 | 10 | 11 | 12 | 13 | 14 |
| --- | --- | --- | --- | --- | --- | --- | --- | --- | --- | --- | --- | --- | --- | --- |
| diploblastic  metazoans | 0 | 0 | 0 | 0 | 0 | 0 | 0 | 0 | 0 | 0 | 0 | 0 | - | 0 |
| Aphroditcaea | 1 | 1 | 1 | 1 | 1 | 0 | 1 | 1 | P | 1 | 1 | 1 | 1 | 1 |
| *Canadia* | 1 | 1 | 0 | 0 | 0 | 1 | 0 | 1 | 0 | 0 | 0 | 0 | - | 0 |
| Canalipalpata | 1 | 1 | 0 | 0 | 0 | 0 | 0 | 1 | 0 | 0 | 0 | 0 | - | 1 |
| Chrysopetalidae | 1 | 1 | 1 | 1 | 1 | 0 | 1 | 1 | 1 | 1 | 1 | 1 | 1 | 1 |
| Eunicida | 1 | 1 | 1 | 1 | 0 | 1 | 1 | 1 | 1 | 1 | 1 | 1 | 0 | 1 |
| Other phyllodocida | 1 | 1 | 1 | 1 | 1 | 0 | 1 | 1 | 1 | 1 | 1 | 1 | 1 | 1 |
| *Pygocirrus* | 1 | ? | ? | ? | ? | ? | ? | 1 | 0 | 0 | 0 | 0 | ? | 1 |
| *Phragmochaeta* | 1 | ? | ? | ? | 0 | ? | 0 | 1 | 0 | 0 | 0 | 0 | - | 0 |
| *Guanshanchaeta* | 1 | 1 | 0 | 0 | 0 | ? | 0 | 1 | 0 | ? | 0 | 0 | - | ? |
| *Burgessochaeta* | 1 | 1 | 0 | 0 | 0 | 1 | 0 | 1 | 0 | 0 | 0 | 0 | - | 0 |
| *Insolicorypha* | 1 | ? | 0 | 0 | 0 | ? | 0 | 1 | 0 | 0 | ? | ? | 0 | 0 |
| *Peronochaeta* | 1 | ? | 0 | 0 | 0 | ? | 0 | 1 | 0 | 0 | 0 | 0 | - | 0 |
| *Stephenoscolex* | 1 | ? | ? | ? | 0 | ? | 0 | 1 | 0 | 0 | 0 | 0 | - | 0 |
| *Bundenbachochaeta* | 1 | ? | ? | ? | 0 | ? | 0 | 1 | 1 | 1 | 0 | 0 | - | 0 |

Cladogram: Bootstrap 50% majority-rule consensus tree


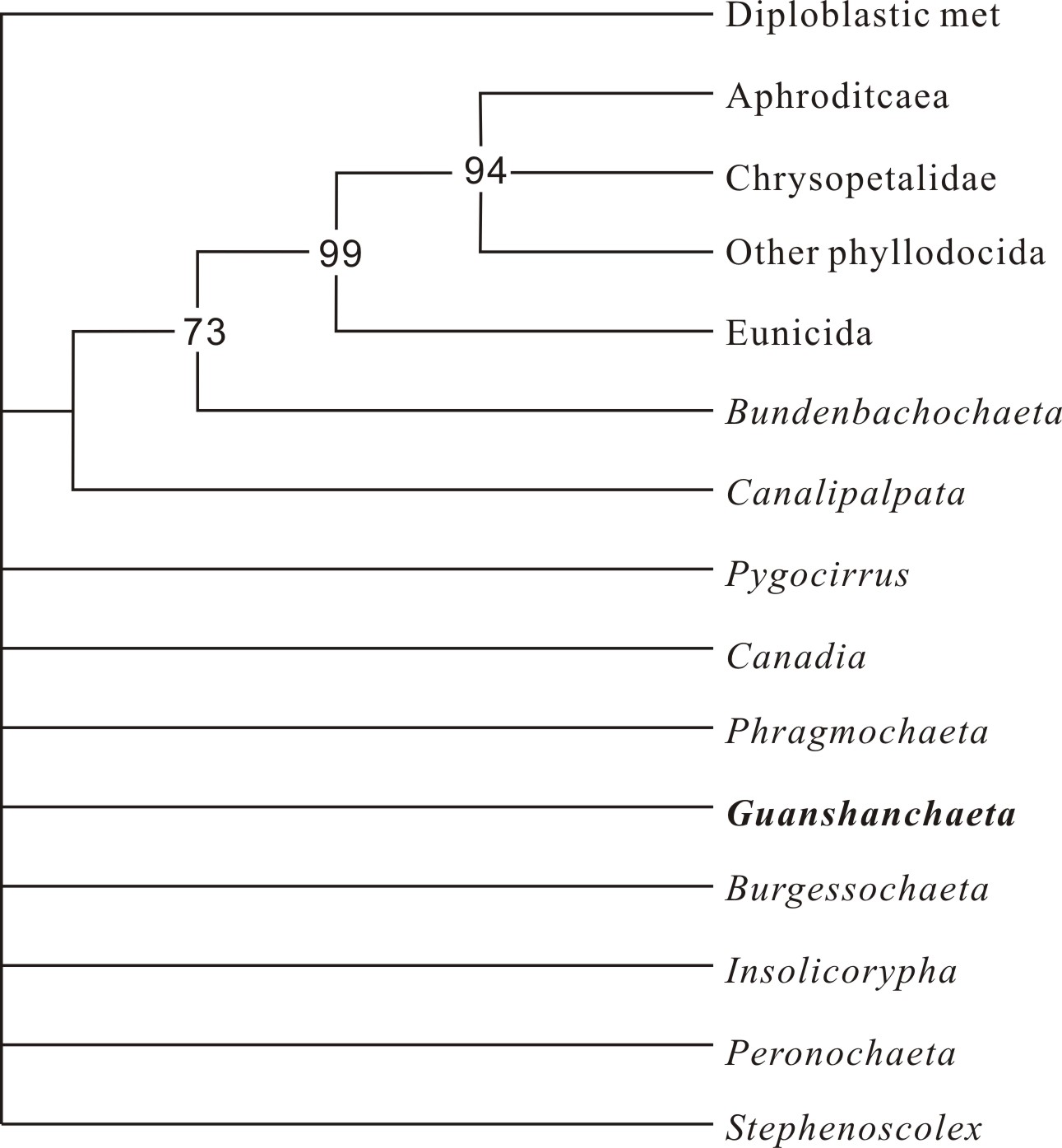


Homoplasy index(HI)=0.1176

CI(excluding uninformative characters)=0.8571

HI(excluding uninformative characters)=0.1429

Retention index(RI)= 0.9375

Rescaled consistency index (RC)=0.8272

nreps=1000
